# How Facial Attractiveness Affects Time Perception: Increased Arousal Results in Temporal Dilation of Attractive Faces

**DOI:** 10.3389/fpsyg.2021.784099

**Published:** 2021-12-10

**Authors:** Sihong Zhou, Lingjing Li, Fuyun Wang, Yu Tian

**Affiliations:** ^1^Institute of Brain and Psychological Sciences, Sichuan Normal University, Chengdu, China; ^2^Sichuan Conservatory of Music, Chengdu, China; ^3^China Academy of Information and Communications Technology, Beijing, China

**Keywords:** time perception, arousal, mediation analysis, automatic suppression, facial attractiveness

## Abstract

Time perception plays a fundamental role in people’s daily life activities, and it is modulated by changes in environmental contexts. Recent studies have observed that attractive faces generally result in temporal dilation and have proposed increased arousal to account for such dilation. However, there is no direct empirical result to evidence such an account. The aim of the current study, therefore, was to clarify the relationship between arousal and the temporal dilation effect of facial attractiveness by introducing a rating of arousal to test the effect of arousal on temporal dilation (Experiment 1) and by regulating arousal *via* automatic expression suppression to explore the association between arousal and temporal dilation (Experiment 2). As a result, Experiment 1 found that increased arousal mediated the temporal dilation effect of attractive faces; Experiment 2 showed that the downregulation of arousal attenuated the temporal dilation of attractive faces. These results highlighted the role of increased arousal, which is a dominating mechanism of the temporal dilation effect of attractive faces.

## Introduction

The ability to perceive time is crucial to people’s everyday life. It is not only fundamental to motor activities (e.g., walking, dancing, and driving) but also essential for social communications (such as word segmentation, speaking speed, and even language understanding). However, unlike the linear progression of physical time, human time perception is not always stable. It can be adaptively distorted by the changing environment rather than perceived veridically.

Recent empirical evidence has suggested that people’s time perception varies with facial attractiveness. Specifically, Ogden (2013) asked participants to verbally estimate the duration of the attractive, average, and unattractive faces. She found that participants’ estimations of the attractive and average faces were longer than those of unattractive faces. Arantes et al. (2013) adopted the temporal reproduction task and found that participants reproduced longer durations for attractive faces and neutral stimuli than unattractive faces. Using a similar temporal reproduction task, Tian et al. (2019) also found the time perception of attractive faces to be longer than that of unattractive faces, and such a temporal dilation effect was highlighted by opposite-sex faces. Tomas and Španić (2016) employed a temporal bisection task that required participants to distinguish whether the presentation time of faces (400–1,600 ms) was close to the short or long anchor (400 vs. 1,600 ms). They found that participants tended to distinguish angry expressions as “long” compared with neutral expressions, but this tendency was salient only under the attractive condition but not the unattractive condition. Taken together, although researchers used different tasks and their designs varied in details, the abovementioned empirical studies generally showed that facial attractiveness influences time perception, i.e., attractive faces tend to result in the dilation effect of time perception.

In the domain of time perception, the most dominant theoretical account in explaining such temporal distortion is the pacemaker-accumulator (PA) models, which posit three main components in generating time perception, involving the pacemaker component, the switch/gate component, and the accumulator component. During timing, the pacemaker emits pulses, which go through the switch/gate to be accumulated by the accumulator. The number of these accumulated pulses then represents people’s time perception (Treisman, 1963; Church, 1984; Gibbon et al., 1984). Based on PA models, time perception can be distorted by two main mechanisms: the rate of the pacemaker and the state of the switch/gate. In a time perception study, pacemaker rate is conceptualized as arousal, with increasing arousal equivalent to an acceleration of the pacemaker (Mella et al., 2011; Lake et al., 2016; Piovesan et al., 2018; Vallet et al., 2019). When the arousal increases, the pacemaker runs faster, more pulses are emitted, thus time is perceived to last longer. Meanwhile, the functionality of the switch/gate is generally conceptualized as the allocation of attention, that is, pulses pass when attention is allocated toward timing, while the pulses are blocked when attention is distracted away from timing, thus the deficit of attention toward timing leads to temporal underestimation (Zakay and Block, 1996; Brown, 1997; Lejeune, 1998; Bar-Haim et al., 2010).

According to PA models, Ogden (2013) attributed the temporal dilation effect of facial attractiveness to attention. Ogden suggested that the time perception of unattractive faces was underestimated in comparison with the attractive and average faces because unattractive faces detracted attention from timing. Alternatively, other researchers suggested that the arousal mechanism should account for the temporal dilation effect of facial attractiveness. Specifically, Arantes et al. (2013) indicated that attractive faces (particularly opposite-gender faces) are biologically salient events that activate the appetitive motivational system, thereby increasing arousal, leading to an overestimation of time perception. Tomas and Španić (2016) proposed that the same-gender attractive faces may activate the defensive motivational system due to potential competition and also result in increased arousal, leading to temporal dilation. Although previous studies have found that attractive faces capture attention earlier than unattractive faces (Maner et al., 2003; Valuch et al., 2015), which may make the switch/gate work earlier and cause temporal dilation (Grommet et al., 2011; Liu et al., 2015), Tian et al. (2019) adopted the reproduction task and pointed out that the temporal dilation effect of attractive faces seems to be more closely associated with increased arousal, suggesting that the arousal mechanism may dominate the effect of facial attractiveness on time perception. They found that male and female participants showed different patterns in perceiving durations of opposite-/same-gender faces. For opposite-gender faces, male and female participants both perceived attractive faces to be longer than unattractive ones, but for same-gender faces, only female participants consistently showed this temporal dilation effect of facial attractiveness. These results have extended the appetitive motivation theory (Arantes et al., 2013), but not the defensive motivation theory (Tomas and Španić, 2016) of arousal mechanism to male participants, suggesting that male participants’ temporal dilation effect of opposite-gender facial attractiveness can be more clearly explained by arousal unidirectionally.

So far, the generally used theoretical explanation of the arousal mechanism underlying the temporal dilation effect of facial attractiveness (Arantes et al., 2013; Tomas and Španić, 2016; Tian et al., 2019) still lacks direct empirical support. The goal of the current study, therefore, was to explore the relationship between arousal and the effect of facial attractiveness on time perception by (1) introducing a subjective rating of arousal and (2) regulating arousal by an emotion regulation paradigm.

Previous studies on time perception have tried to demonstrate the relationship between the increase in arousal and temporal dilation by testing the association between the measured arousal and time perception. Common ways to manipulate arousal include employing experimental materials in different arousal levels from a standardized emotional system and dividing arousing manipulations into corresponding categorizations. For example, Mella et al. (2011) employed neutral, low-arousal negative, and high-arousal negative sounds from the International Affective Digitalized Sounds System as stimuli and adopted the skin conductance response (SCR) as an indicator of arousal. They found that a higher level of arousal generates longer time perception (Mella et al., 2011). Fayolle et al. (2015) used electric shocks (shock vs. no-shock) to manipulate arousal and adopted self-report ratings and SCR to assess arousal. They observed that the temporal dilation effect of electric shocks increased with an increase in arousal (Fayolle et al., 2015). However, the relationship between facial attractiveness and arousal has been evidenced to be non-linear. Specifically, attractive faces are more arousing than the average and unattractive faces, but unattractive faces are also generally more arousing than average faces. As our goal was to explore the relationship between arousal and the temporal dilation effect of facial attractiveness, it seems difficult to achieve our goal by dividing experimental materials with arousal. Alternatively, we intended to employ faces with increasing facial attractiveness (ranging from unattractive to attractive) as experimental materials, measure the participants’ time perception and arousal to these face stimuli, and use the mediating effect analysis to test the hypothesis that increasing arousal mediates the temporal dilation effect of facial attractiveness (**Hypothesis 1**).

With the second goal to regulate arousal, we chose to study time perception in the framework of emotion regulation. According to the process model of emotion regulation (Gross, 1998), five major emotion regulation strategies can be used at each of the many steps in the process through emotion-generation: situation selection, situation modification, attention allocation, cognitive reappraisal, and expression suppression. Among them, expression suppression is a strategy directed toward inhibiting behaviors associated with emotional responding (e.g., facial expressions, verbal utterances, and gestures), and has been proven to be effective for reducing arousal (Eippert et al., 2007; Goldin et al., 2008; Flynn et al., 2010; Yuan et al., 2015; Cai et al., 2016). Importantly, by requiring participants to deliberately suppress their expression, previous studies have successfully eliminated the emotional temporal dilation effect and attributed such elimination to the reduction of arousal (Effron et al., 2006; Tian et al., 2018). However, as deliberately suppressing expression is a costly strategy in terms of attentional resources (Gross, 1998; Ohira et al., 2006; Yuan et al., 2015), and might therefore lead to a temporal underestimation due to deficits in attention (Zakay, 1989; Zakay and Block, 1997), the use of deliberate suppression may mix the mechanisms of arousal and attention. Fortunately, some evidence has shown that expression suppression can be conducted automatically *via* a priming task that passively activates the goal of suppression and then realizes this goal without one’s awareness (Bargh et al., 2001). Such automatic suppression has been shown to consume few attentional resources (Mauss et al., 2007a,b; Gallo et al., 2009), and to effectively reduce arousal (Mauss et al., 2007b; Chen et al., 2017a). Considering this, we intended to adopt automatic rather than deliberate expression suppression to regulate arousal and hypothesized that the temporal dilation effect of facial attractiveness would be attenuated by the manipulation of automatic expression suppression (**Hypothesis 2**).

Consequently, the current study has two aims: to explore the role of arousal in the effect of facial attractiveness on time perception by testing the mediating effect of arousal on temporal dilation (Experiment 1) and to regulate arousal by automatic expression suppression to explore the association between arousal and temporal dilation (Experiment 2). A temporal reproduction task would be employed to measure time perception. As previous studies have found that the arousal effect is most salient at 2,000 ms (Bar-Haim et al., 2010; Liu and Li, 2019), the current study adopted 2,000 ms as the target duration. Meanwhile, a self-reported 9-point scale was adopted to assess the facial attractiveness and arousal of each face. A sentence-unscrambling task that features suppression-related words (Mauss et al., 2007b; Yang et al., 2014; Yuan et al., 2020) was used to prime automatic expression suppression. As both physical and psychological gender have been found to have impacts on facial attractiveness (Samson and Janssen, 2014; Tian et al., 2019), all stimuli were female faces. Accordingly, heterosexual men were recruited as participants. All face stimuli were in neutral expressions to prevent a possible confounding effect of facial expressions.

## Experiment 1

### Method

#### Participant

An *a priori* power analysis was adopted using the G*Power software to determine the sample size (Faul et al., 2007). The effect size was set to a threshold of medium (i.e., 0.25) concerning previous studies using the duration reproduction task (Arantes et al., 2013; Tian et al., 2019), the alpha was set to 0.05, and power was set to 0.8. The results indicated that 28 participants are sufficient for Experiment 1.

Forty healthy men were recruited from a Chinese university. Their ages range from 18 to 24 (*M* ± SD = 20.63 ± 1.81) years. They had a normal or corrected-to-normal vision and were right-handed as assessed by the Edinburgh Handedness Inventory (Oldfield, 1971). They were self-reported heterosexual, and none reported a history of neurological or psychiatric disorders. All participants received a moderate payment for their participation. The experiment was approved by the Ethical Committee of Human Research at Sichuan Normal University.

#### Stimuli

Twenty-four color images of female faces (four high-unattractive, four medium-unattractive, four low-unattractive, four low-attractive, four medium-attractive, and four high-attractive) were selected for Experiment 1. All the face stimuli displayed neutral expressions with full frontal views and were unified into a white background within 320 pixels × 400 pixels. The images were selected and categorized into each condition by the experimenters. To validate stimuli manipulations, these faces were rated by participants in attractiveness and arousal *via* a 9-point scale in the rating part of the experiment (see Table 1).

**TABLE 1:** Mean (SD) of attractiveness and arousal for the faces in Experiment 1 (attractiveness: from 1 = “extremely unattractive” to 9 = “extremely attractive”; arousal: from 1 = “not excited at all” to 9 = “extremely excited”).

	High-unattractive	Medium-unattractive	Low-unattractive	Low-attractive	Medium-attractive	High-attractive
Attractiveness rating	3.01 (0.29)	4.15 (0.33)	4.87 (0.35)	5.17 (0.31)	6.16 (0.46)	7.48 (0.36)
Arousal rating	4.92 (0.59)	3.91 (0.59)	3.01 (0.48)	3.07 (0.61)	5.18 (0.63)	6.43 (0.52)

A repeated-measure ANOVA of attractiveness ratings with Attractiveness Polarity (two levels: attractive vs. unattractive) and Attractiveness Strength (three levels: high, medium, and low) showed a significant main effect of Attractiveness Polarity, *F*(1,39) = 2,312.35, *p* < 0.001, *η*_*p*_^2^ = 0.98, attractive faces were rated as systematically more attractive than unattractive faces. This main effect was modulated by Attractiveness Strength, as revealed by a significant interaction between these factors, *F*(2,78) = 695.35, *p* < 0.001, *η*_*p*_^2^ = 0.95. The simple effects analysis showed that in the attractive face session, the high-attractive faces were rated as more attractive than the medium- or low-attractive faces (*p*s < 0.001), the medium-attractive faces were also rated as more attractive than the low-attractive faces (*p* < 0.001); in the unattractive face session, the high-unattractive faces were rated as less attractive than the medium- or low-unattractive faces (*p*s < 0.001), the medium-unattractive faces were also rated as less attractive than the low-unattractive faces (*p* < 0.001).

A similar repeated-measure ANOVA of arousal ratings showed a significant main effect of Attractiveness Polarity, *F*(1,39) = 184.18, *p* < 0.001, *η*_*p*_^2^ = 0.83, attractive faces were rated as systematically more arousing than unattractive faces. This main effect was modulated by Attractiveness Strength, as revealed by a significant interaction between these factors, *F*(2,78) = 43.41, *p* < 0.001, *η*_*p*_^2^ = 0.53. The simple effects analysis showed that in the attractive face session, the high-attractive faces were rated as more arousing than the medium- or low-attractive faces (*p*s < 0.001), the medium-attractive faces were also rated as more arousing than the low-attractive faces (*p* < 0.001); in the unattractive face session, the high-unattractive faces were rated as more arousing than the medium- or low-unattractive faces (*p*s < 0.001), the medium-unattractive faces were also rated as more arousing than the low-unattractive faces (*p* < 0.001).

#### Procedure

Participants were tested individually in a quiet and dimly lit room. They were required to seat approximately 60 cm from a 17″ LED screen (1,024 pixels × 768 pixels, 60 Hz) with horizontal and vertical visual angles of less than 16° and give their responses *via* a computer keyboard. The whole experiment involved two parts: the temporal reproduction task and the assessments of facial attractiveness and arousal. Two experimental parts were counterbalanced across participants.

In the part of the temporal reproduction task, each trial started with a fixation cross for 500–750 ms. It was immediately followed by a face, which was presented for 1,000, 2,000, or 3,000 ms. Subsequently, a question mark appeared on the screen cueing the participant to reproduce the time of face presentation. The question mark would remain on the screen either for 3,000 ms or until the participant responded by pressing the spacebar for a duration equivalent to the time the face was presented. An image of a pink oval with a white background appeared at the beginning of the key press and remained until the participant released the spacebar. Lastly, a 1,000 ms blank was presented. To ensure that the trials of target duration were about 80% (Li et al., 2017, 2019), each image was presented with eight repetitions at 2,000 ms, and only once at 1,000 and 3,000 ms, for a total of 240 trials. The trial order was randomized across participants.

**FIGURE 1:**
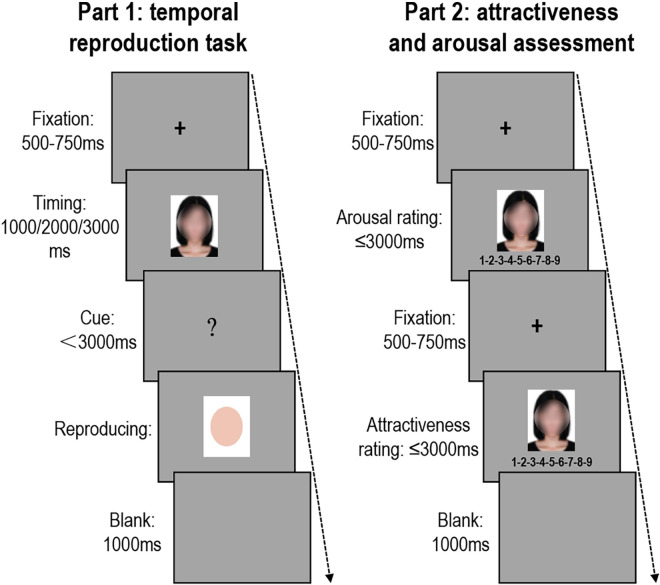
The procedure of Experiment 1. Schematic illustration of the temporal reproduction task **(left panel)** and schematic illustration of the attractiveness and arousal assessment **(right panel)**. The faces were shown as blurred ones here for privacy.

In the rating part, participants were instructed to complete the assessments of attractiveness and arousal *via* a 9-point rating scale. Each trial started with a fixation cross for 500–750 ms. Then, a face was presented, in which participants were required to, respectively, rate its attractiveness and arousal within 3,000 ms, following a 1,000 ms blank (see Figure 1). Each face was assessed for attractiveness and arousal for once, which means a total of 24 trials. The trial order was randomized across participants, and the order of facial attractiveness and arousal assessments were counterbalanced across participants.

### Results

#### The Effect of Facial Attractiveness on Time Perception

A repeated-measures ANOVA of reproduced duration with Attractiveness Polarity (two levels: attractive vs. unattractive) and Attractiveness Strength (three levels: high, medium, and low) revealed a significant main effect of Attractiveness Polarity, *F*(1,39) = 156.60, *p* < 0.001, *η*_*p*_^2^ = 0.80, attractive faces were reproduced as systematically longer than unattractive faces. This main effect was modulated by Attractiveness Strength, as revealed by a significant interaction between these factors, *F*(2,78) = 68.80, *p* < 0.001, *η*_*p*_^2^ = 0.64. The simple effects analysis showed that in the attractive face session, the high-attractive faces were reproduced as longer than the medium- or low-attractive faces (*p*s < 0.001), the medium-attractive faces were also reproduced as longer attractive than the low-attractive faces (*p* < 0.001); in the unattractive face session, the high-unattractive faces were reproduced as longer attractive than the medium- or low-unattractive faces (*p*s < 0.001), the medium-unattractive faces were also reproduced as longer attractive than the low-unattractive faces (*p* < 0.001). Further comparison found that for high and medium strength faces, the temporal dilation effects in the attractive face session was greater than those in the unattractive face session (*ps* < 0.001).

**FIGURE 2:**
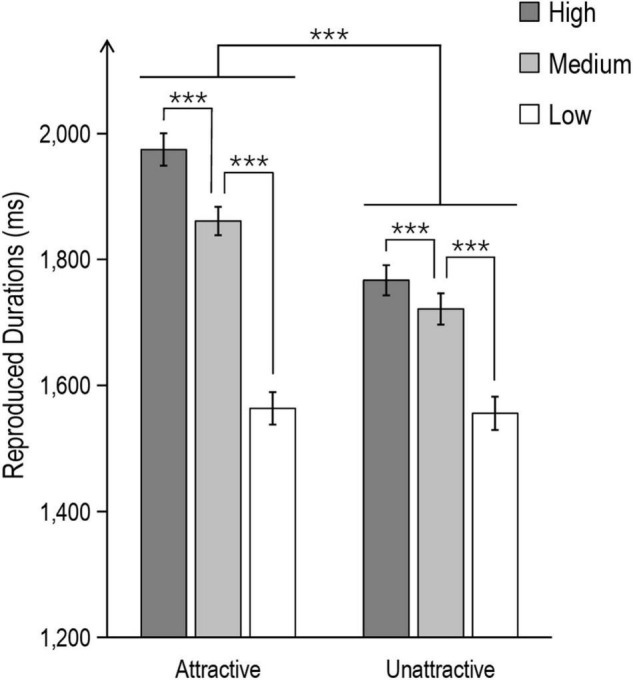
Mean reproduced durations for each condition in Experiment 1. The error bar represents the SE (significance level ***p < 0.001).

These results suggested that facial attractiveness could modulate time perception. Specifically, both increase and decrease of facial attractiveness led to the dilation of time perception, and attractive faces tended to result in salient temporal dilation (see Figure 2).

#### The Mediating Effect of Arousal on Temporal Dilation

The *a priori* test revealed that the relationship between facial attractiveness and arousal fitted the U curve. Specifically, the closer the attractiveness rating was to the polarity (i.e., unattractive = 1 and attractive = 9), the higher the rating of arousal was. Therefore, we divided the polarity of attractiveness into attractive and unattractive sessions, and then performed mediation analyses to examine whether the relationship between facial attractiveness (X = attractiveness ratings) and time perception (Y = reproduced durations) was mediated by arousal (M = arousal ratings). The mediation analysis was performed with the PROCESS macro developed by Hayes (2013).

**FIGURE 3:**
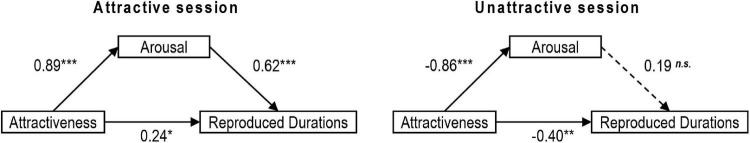
Mediation models in Experiment 1. Mediation model for the attractive session **(left panel)** and mediation model for the unattractive session **(right panel)**. The values represent the standardized regression coefficients (β) for the direct and indirect effects of Attractiveness on Reproduced Durations (significance level ***p < 0.001; **p < 0.01; *p < 0.05; *^n.s.^p* > 0.05). Solid lines are used for significant effects, and dashed lines are used for insignificant effects.

In the attractive session, the results showed that the 95% CI of an indirect effect of X on Y excluded 0, 95% CI: 63.12, 144.60, *Effect* = 103.08, *BootSE* = 20.31, the total effect of X on Y was significant, *Effect* = 148.91, SE = 10.60, *t* = 14.05, *p* < 0.001, 95% CI: 127.92, 169.89, and the direct effect of X on Y was significant, *Effect* = 45.83, SE = 20.69, *t* = 2.22, *p* < 0.05, 95% CI: 4.85, 86.79, thus indicating that arousal acted as a mediator between facial attractiveness and time perception in the attractive session; however, in the unattractive session, the results showed that the 95% CI of an indirect effect of X on Y included 0, 95% CI: −71.17, 15.08, suggesting that the mediating effect of arousal did not reach significance in the unattractive session (see Figure 3).

### Discussion

In Experiment 1, we adopted subjective arousal ratings to test the hypothesis that increasing arousal mediates the temporal dilation effect of facial attractiveness (**Hypothesis 1**). The results showed that increasing arousal mediated the temporal dilation effect of attractive faces, suggesting that the increased arousal was the mechanism of the temporal dilation effect of attractive faces.

However, this underlying mechanism needs to be more directly tested. Therefore, in Experiment 2, we tried to regulate the arousal level and check whether this regulation can lead to changes in the temporal dilation effect. Again, we adopted the mediation analysis to illustrate the relationship between automatic expression suppression, arousal, and the temporal dilation effect.

## Experiment 2

### Method

#### Participants

The *a priori* power analysis was the same as the one used in Experiment 1. The results indicated that 28 participants are sufficient for Experiment 2.

A new cohort of 60 participants from the same Chinese university was recruited for Experiment 2. They were randomly assigned to one of the two groups: an experimental group (age: 18–23 years, *M* ± SD = 20.37 ± 1.77 years) and a control group (age: 18–24 years, *M* ± SD = 20.46 ± 1.93). All reported to have a normal or corrected-to-normal vision and were right-handed as assessed by the Edinburgh Handedness Inventory (Oldfield, 1971). They were self-reported heterosexual, and none reported a history of neurological or psychiatric disorders. All participants received a moderate payment for their participation. The experiment was approved by the Ethical Committee of Human Research at Sichuan Normal University.

#### Stimuli

Twenty-four color images of female faces were used as stimuli, including eight attractive, eight average, and eight unattractive faces. All the faces displayed neutral expressions with full frontal views and were unified into a white background within 320 pixels × 400 pixels.

Images were tested using the results of a prior stimulus rating in which 30 additional male participants (age: 18–24 years, *M* ± SD = 20.23 ± 1.99) from the same Chinese university rated the attractiveness of 24 female faces *via* a 9-point rating scale (from 1 = “extremely unattractive” to 9 = “extremely attractive”). All raters reported to have a normal or corrected-to-normal vision and were right-handed. They were self-reported heterosexual, none reported a history of neurological or psychiatric disorders, and can be considered as homogeneous to participants in Experiments 1 and 2. These faces were rated by the rater group in attractiveness, arousal *via* a 9-point scale (see Table 2).

**TABLE 2:** Mean (SD) of attractiveness for the faces in Experiment 2 (attractiveness: from 1 = “extremely unattractive” to 9 = “extremely attractive”).

	Unattractive face	Average face	Attractive face
Attractiveness rating	3.06 (0.29)	5.04 (0.35)	7.21 (0.39)

A repeated-measures ANOVA with Attractiveness (three levels: attractive, average, and unattractive) confirmed significant differences in the attractiveness ratings for the three conditions, *F*(2,58) = 1,166.05, *p* < 0.001, *η*_*p*_^2^ = 0.98. *Post hoc* tests (Bonferroni corrected) showed that attractive faces were rated as more attractive than the average or unattractive faces (*p*s < 0.001), and that average faces were also rated as more attractive than unattractive faces (*p* < 0.001).

#### Procedure

The procedure for Experiment 2 involved three sequential parts: a sentence-unscrambling task, a temporal reproduction task, and an arousal assessment.

**FIGURE 4:**
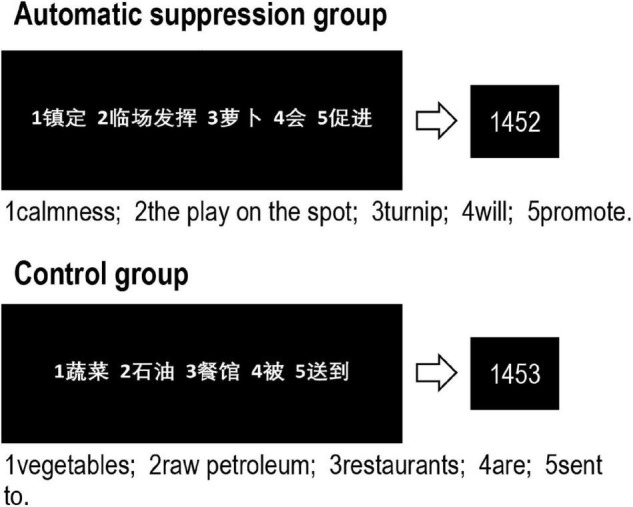
Schematic illustration of the sentence-unscrambling task for the automatic suppression group **(top panel)** and control group **(bottom panel)**.

The sentence-unscrambling task was used to prime participants of suppression automatically. It was first adapted to manipulate the automatic emotion regulation by Mauss et al. (2007a,b). In this task, participants are required to construct grammatically correct, four-word sentences from five-word jumbles. For the experimental group, there were 20 sentences each included an “emotion suppression” term; for the control group, there were 20 sentences each included a “passively perceiving” term (see Figure 4). These terms were selected from a previous study (Yuan et al., 2020), and were effective in priming suppression and passively perceiving.

The time reproduction task and arousal assessment in Experiment 2 are similar to Experiment 1, except that different stimuli were used. In the time reproduction task, to ensure that the trials of target duration were about 80% (Li et al., 2017, 2019), each image was presented with eight repetitions at 2,000 ms, and only once at 1,000 and 3,000 ms, for a total of 240 trials; in the arousal assessment, each face was assessed for arousal for once, which means a total of 24 trials. The trial order was randomized across participants.

At the end of Experiment 2, participants were required to complete a funneled debriefing procedure, which is similar to previous studies (Chartrand and Bargh, 1996; Bargh and Chartrand, 2000; Williams et al., 2009; Yuan et al., 2020). They were asked (1) whether they had ever seen or completed a sentence-unscrambling task for another experiment, (2) what they thought about the purpose of the sentence-unscrambling task, and (3) whether and what they thought that the sentence-unscrambling task and the temporal reproduction task had been related. This funneled debriefing procedure was used to check the validity of the automatic suppression manipulation. For the first question, nobody reported they had seen or completed a sentence-unscrambling task before. For the second question, most participants (*n* = 58) believed that the purpose of the sentence-unscrambling task was to test their grammatical abilities, and a few participants (*n* = 2) reported that they did not know. For the third question, more than a half of participants (*n* = 34) thought that the sentence-unscrambling task had nothing to do with the temporal reproduction task, and the remaining participants thought that the sentence-unscrambling task may have some relationship with the temporal reproduction task (*n* = 26), but nobody mentioned emotion or emotion regulation as the connection between the two tasks. Thus, no participants indicated suspicion of the experimental manipulation.

### Result

#### Manipulation Check of Temporal Dilation

The control group data were used to check the temporal dilation effect of facial attractiveness. A repeated-measures ANOVA with Attractiveness (three levels: attractive, average, and unattractive) confirmed significant differences in the reproduced duration for the three conditions, *F*(2,58) = 1,938.27, *p* < 0.001, *η*_*p*_^2^ = 0.99. *Post hoc* tests (Bonferroni corrected) showed that attractive faces were reproduced as longer than the average or unattractive faces (*p*s < 0.001), unattractive faces were reproduced as longer than average faces (*p* < 0.001), suggesting that both attractive and unattractive faces led to the dilation of time perception, while attractive faces resulted in greater temporal dilation.

#### The Effect of Suppression on Arousal

A mixed-design ANOVA of arousal ratings with Attractiveness (three levels: attractive, average, and unattractive) as a within-subject factor and Group (two levels: control and experimental) as a between-subject factor revealed a significant main effect of Attractiveness, *F*(2,116) = 899.69, *p* < 0.001, *η*_*p*_^2^ = 0.94. *Post hoc* tests (Bonferroni corrected) showed that attractive faces were systematically rated as more arousing than average (*p* < 0.001), and unattractive faces were also systematically rated as more arousing than average (*p* < 0.001). The main effect of Group was also significant, showing that the experimental group systematically rated less arousal than the control group, *F*(1,58) = 935.19, *p* < 0.001, *η*_*p*_^2^ = 0.94. Importantly, the ANOVA found a significant interaction of Attractiveness by Group, *F*(2,116) = 175.98, *p* < 0.001, *η*_*p*_^2^ = 0.75. Further comparisons found that in the control group, the arousal effect of attractive faces is significantly greater than that of unattractive faces, *p* < 0.001; in contrast, in the experimental group, the arousal effect between attractive faces and unattractive faces was non-significant, *p* > 0.05.

These results indicated that the manipulation of automatic expression suppression attenuated the arousal rating for both attractive and unattractive faces, and the attenuation was salient for attractive faces.

#### The Effect of Suppression on Temporal Dilation

**FIGURE 5:**
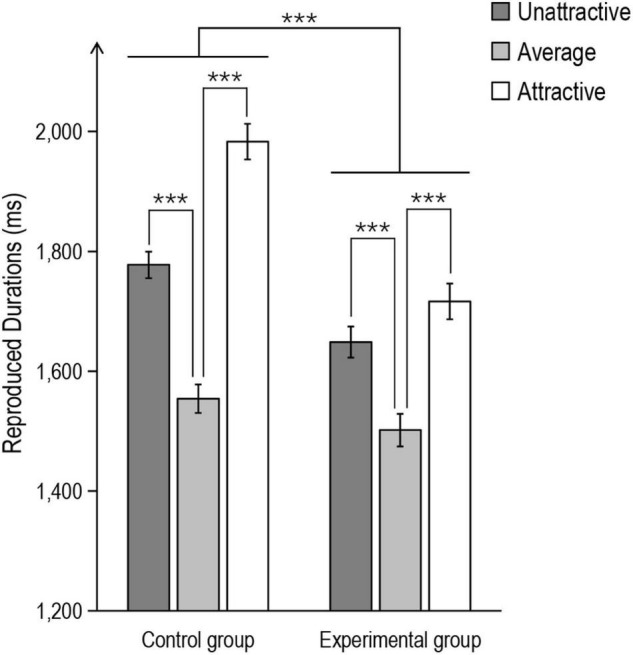
Mean reproduced durations for each condition in Experiment 2. The error bar represents the SE. (significance level ***p < 0.001).

A mixed-design ANOVA of reproduced duration with Attractiveness (three levels: attractive, average, and unattractive) as a within-subject factor and Group (two levels: control and experimental) as a between-subject factor revealed a significant main effect of Attractiveness, *F*(2,116) = 2,586.44, *p* < 0.001, *η*_*p*_^2^ = 0.98. *Post hoc* tests (Bonferroni corrected) showed that attractive faces were systematically reproduced as longer than average faces (*p* < 0.001), and unattractive faces were also reproduced as longer than average faces (*p* < 0.001). The main effect of Group was also significant, showing that the experimental group systematically reproduced a shorter duration than the control group, *F*(1,58) = 1,835.47, *p* < 0.001, *η*_*p*_^2^ = 0.97. Importantly, the ANOVA found a significant interaction of Attractiveness by Group, *F*(2,116) = 291.93, *p* < 0.001, *η*_*p*_^2^ = 0.83. Further comparison found that the temporal dilation in the experimental group was generally smaller than that in the control groups, *p*s < 0.05 (see Figure 5).

These results indicated that the manipulation of automatic expression suppression attenuates the temporal dilation of both attractive and unattractive faces, and the attenuation is greater for attractive faces, suggesting that arousal plays an important role in the temporal dilation effect of attractive faces.

#### The Mediating Effect of Arousal on Temporal Dilation

In line with Experiment 1, we divided the polarity of attractiveness into attractive and unattractive conditions, then performed mediation analyses to explore the role of arousal in the temporal dilation of facial attractiveness. The Group was X (control group = 0, experimental group = 1), the Temporal Difference was Y (mean attractive/unattractive – mean average, for each participant), and Arousal Difference (mean attractive/unattractive – mean average, for each participant) was M.

**FIGURE 6:**
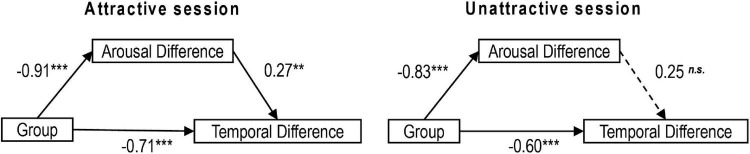
Mediation models in Experiment 2. Mediation model for the attractive session **(left panel)** and mediation model for the unattractive session **(right panel)**. The values represent the standardized regression coefficients (β) for the direct and indirect effects of Attractiveness on Reproduced Durations (significance level ***p < 0.001; **p < 0.01; ^n.s.^*p* > 0.05). Solid lines are used for significant effects, and dashed lines are used for insignificant effects.

In the attractive session, the results showed that the 95% CI of an indirect effect of X on Y excluded 0, 95% CI: −97.70, −7.6, *Effect* = −54.64, *BootSE* = 23.13, the total effect of X on Y was significant, *Effect* = −213.98, SE = 9.13, *t* = −23.43, *p* < 0.001, 95% CI: −232.26, −195.71, and a direct effect of X on Y was significant, *Effect* =−159.35, SE = 21.40, *t* = −7.44, *p* < 0.001, 95% CI: −202.20, −116.49, indicating that arousal acted as a mediator between automatic expression suppression and temporal dilation in the attractive session; however, in the unattractive session, the results showed that the 95% CI of an indirect effect of X on Y included 0, 95% CI: −41.46, 1.30, suggesting that the mediating effect of arousal did not reach significance in the unattractive session (see Figure 6).

These results highlighted that the decrease of arousal acted as a mediator between automatic expression suppression and temporal dilation in the attractive session, indicating that arousal is the mechanism of the temporal dilation effect of attractive faces.

### Discussion

In Experiment 2, we used the manipulation of automatic expression suppression, i.e., the sentence-unscrambling task including “emotion suppression” terms, to test the hypothesis that the temporal dilation effect of facial attractiveness would be attenuated by the manipulation of automatic expression suppression (**Hypothesis 2**). The results showed that arousal mediated the effect of the automatic suppression manipulation on the temporal dilation of attractive faces (but of unattractive faces), adding direct empirical evidence to the arousal mechanism underlying the temporal dilation effect of facial attractiveness.

## General Discussion

Recent studies have shown that attractive faces generally result in temporal dilation (Arantes et al., 2013; Ogden, 2013; Tomas and Španić, 2016; Tian et al., 2019), and most researchers proposed that increased arousal evoked by attractive faces should account for the temporal dilation effect of facial attractiveness (Arantes et al., 2013; Tomas and Španić, 2016; Tian et al., 2019). The goal of the current study was therefore to verify such an account with two empirical experiments. Experiment 1 aimed at introducing a rating of arousal to test the mediating effect of arousal between facial attractiveness and temporal dilation. Experiment 2 focused on regulating arousal *via* automatic expression suppression to explore the association between arousal and temporal dilation.

In both Experiments 1 and 2, the current study convergently observed that facial attractiveness influences time perception. Specifically, the results of Experiment 1 showed that in both attractive and unattractive sessions, the closer the attractiveness rating was to attractiveness polarity (i.e., “extremely unattractive” and “extremely attractive”) the longer time was perceived, while the attractive faces tended to result in salient temporal dilation. The results of the control group in Experiment 2 showed that both attractive and unattractive faces led to temporal dilation and the attractive faces resulted in greater temporal dilation. These results revealed that the current study replicated the findings of previous studies that the attractive face tended to result in the dilation effect of time perception (Arantes et al., 2013; Ogden, 2013; Tomas and Španić, 2016; Tian et al., 2019), suggesting that the effect of facial attractiveness on time perception was stable and reliable. Importantly, these results also provided a baseline for exploring the role of arousal in the effect of facial attractiveness on time perception.

Following **Hypothesis 1**, Experiment 1 found that arousal mediated the temporal dilation effect of attractive faces, that is, the increase in facial attractiveness increased the arousal and consequently led to the dilation effect of time perception. In the dominant theoretical accounts, PA models assumed that the time perception is determined according to the number of pulses generated by a pacemaker and counted by an accumulator through a switch/gate (Treisman, 1963; Church, 1984; Gibbon et al., 1984). Based on PA models, the pacemaker rate is usually conceptualized as arousal (Lake et al., 2016; Zhang et al., 2017; Li and Tian, 2020; Yuan et al., 2020), with an increase in arousal equivalent to an increase in the pacemaker rate. This increase in the pacemaker rate results in a greater number of pulses during the timing of a specific event and eventually led to temporal dilation. Anecdotal experience and empirical evidence both showed that attractive faces are arousing, and the more attractive the faces are rated, the more arousing they are (Maner et al., 2003; Nakamura and Kawabata, 2014; Lin et al., 2016). In consequence, previous researchers theoretically attributed the temporal dilation of attractive faces to arousal (Arantes et al., 2013; Tomas and Španić, 2016; Tian et al., 2019), and Experiment 1 directly evidenced such an inference by observing that increasing arousal mediated the dilation effect of time perception in attractive faces.

However, the observed association between increased arousal and temporal dilation only illustrated the role of arousal in time perception on a descriptive level. To further explore the relationship between arousal and the effect of facial attractiveness on time perception, Experiment 2 employed a sentence-unscrambling task that features suppression-related words to manipulate automatic expression suppression. Such manipulation was thought to be able to passively activate the goal of emotion suppression, thereby realizing the goal without the participant’s awareness (Bargh and Chartrand, 2000; Bargh et al., 2001), and it is effective in reducing arousal and consuming little or no attentional resource (Gao et al., 2018). The results of arousal showed that the manipulation of suppression was effective in decreasing arousal, which was in line with previous studies (Mauss et al., 2007b; Chen et al., 2017b). The results of time perception revealed that the manipulation of suppression attenuated temporal dilation. Importantly, as hypothesized (**Hypothesis 2**), the results of mediating analysis of arousal on temporal dilation indicated that the decrease of arousal mediates the attenuation of temporal dilation in attractive faces, suggesting that the manipulation of automatic expression suppression decreases the arousal and consequently attenuates the temporal dilation of attractive faces. This finding is similar to previous investigation concerning emotional temporal dilation (Mella et al., 2011) or anxiety-related temporal dilation (Yuan et al., 2020), providing empirical evidence that the attenuation of temporal dilation was associated with the downregulation of arousal.

Taken together, the results of both Experiments 1 and 2 convergently highlighted the role of arousal in the temporal dilation effect of attractive faces. Specifically, the attractive faces generally induce an increase in arousal and consequently lead to the dilation of time perception; however, when the arousal of attractive faces was downregulated, the dilation of time perception would be correspondingly attenuated. These results suggested that an increase in arousal played a dominating mechanism in the temporal dilation effect of attractive faces. From the perspective of motivation (Peeters and Czapinski, 1990; Yuan et al., 2019), the increased arousal induced by attractive faces reflected the evolutionary activation of the approach motivation system (Rhodes, 2006). Accordingly, the temporal dilation effect of attractive faces could be interpreted as allowing individuals to prepare more for subsequent approaching behavior, rather than a byproduct of the increased arousal. On the other hand, the mediating effect of arousal on temporal dilation reflected the adaptation of time perception. The time perception could be dilated by the increased arousal, and the temporal dilation effect could be also attenuated by the manipulation of downregulating arousal. Such flexibility of time perception reflected the processes that allow individuals to adaptively respond to changes in the environment (Cicchini et al., 2012; Lake et al., 2016).

Several limitations of the current study should be addressed in future work. First, a self-reported 9-point scale was used to assess arousal. Some studies conceptualized the arousal of timing models as physiological measurement indicators (Angrilli et al., 1997; Mauss et al., 2004; Lake et al., 2016) due to its precision (Wilhelm et al., 2001; Edelmann and Baker, 2002). Although self-reporting and physiological results are somewhat consistent (McLeod et al., 1986; Gross, 1998; Kuo and Linehan, 2009), if future studies need to precisely assess arousal, physiological measurements should be used. Second, only male participants were employed. Although a previous study found that the temporal dilation effect of facial attractiveness was equally exhibited in both male and female (Tian et al., 2019), some empirical evidence showed that the emotional susceptibility of female was higher than that of male (Campanella et al., 2004; Yuan et al., 2009). Thus, the current results might underestimate the effect of arousal. Third, the efficacy of suppression is culture-specific: East Asians show better performance than Westerners (Butler et al., 2007; Murata et al., 2013), and suppression has been determined to produce beneficial emotion-regulation effects for Chinese at both behavioral and physiological levels (Yuan et al., 2015). However, all the participants of the current study were Chinese. This means that if future studies should employ Western participants, the arousal attenuation effect found in Experiment 2 may not be repeated.

## Conclusion

The current study aimed to clarify the relationship between arousal and the temporal dilation effect of facial attractiveness. The results highlighted the role of arousal: (1) the increased arousal induced by attractive faces mediated the temporal dilation effect of facial attractiveness and (2) the downregulation of arousal attenuated the temporal dilation of attractive faces. These results indicated that increasing arousal is the dominating mechanism of the temporal dilation effect of attractive faces.

## Data Availability Statement

The original contributions presented in the study are included in the article/Supplementary Material, further inquiries can be directed to the corresponding author.

## Ethics Statement

The studies involving human participants were reviewed and approved by the Ethical Committee of Human Research at Sichuan Normal University. The patients/participants provided their written informed consent to participate in this study.

## Author Contributions

YT designed the experiment. YT and LL acquired and analyzed the data. All authors contributed to the interpretation of the data and approved the final version of the manuscript.

## Conflict of Interest

The authors declare that the research was conducted in the absence of any commercial or financial relationships that could be construed as a potential conflict of interest.

## Publisher’s Note

All claims expressed in this article are solely those of the authors and do not necessarily represent those of their affiliated organizations, or those of the publisher, the editors and the reviewers. Any product that may be evaluated in this article, or claim that may be made by its manufacturer, is not guaranteed or endorsed by the publisher.
